# Simultaneous siRNA Targeting of Src and Downstream Signaling Molecules Inhibit Tumor Formation and Metastasis of a Human Model Breast Cancer Cell Line

**DOI:** 10.1371/journal.pone.0019309

**Published:** 2011-04-26

**Authors:** Jeffrey D. Bjorge, Andy S. Pang, Melanie Funnell, Ke Yun Chen, Roman Diaz, Anthony M. Magliocco, Donald J. Fujita

**Affiliations:** 1 Department of Biochemistry and Molecular Biology, University of Calgary, Calgary, Alberta, Canada; 2 Southern Alberta Cancer Research Institute, University of Calgary, Calgary, Alberta, Canada; 3 Department of Oncology, University of Calgary, Calgary, Alberta, Canada; 4 Department of Pathology and Laboratory Medicine, University of Calgary, Calgary, Alberta, Canada; Beth Israel Deaconess Medical Center, United States of America

## Abstract

**Background:**

Src and signaling molecules downstream of Src, including signal transducer and activator of transcription 3 (Stat3) and cMyc, have been implicated in the development, maintenance and/or progression of several types of human cancers, including breast cancer. Here we report the ability of siRNA-mediated Src knock-down alone, and simultaneous knock-down of Src and Stat3 and/or cMyc to inhibit the neoplastic phenotype of a highly metastatic human model breast cancer cell line, MDA-MB-435S, a widely used model for breast cancer research.

**Methodology/Results:**

Src and its downstream signaling partners were specifically targeted and knocked-down using siRNA. Changes in the growth properties of the cultured cancer cells/tumors were documented using assays that included anchorage-dependent and -independent (in soft agar) cell growth, apoptosis, and both primary and metastatic tumor growth in the mouse tumor model. siRNA-mediated Src knock-down alone, and simultaneous knock-down of Src and Stat3 and/or cMyc inhibited the neoplastic phenotype of a highly metastatic human model breast cancer cell line, MDA-MB-435S. This knock-down resulted in reduced growth in monolayer and soft agar cultures, and a reduced ability to form primary tumors in NOD/SCID mice. In addition, direct intra-tumoral injection of siRNAs targeting these signaling molecules resulted in a substantial inhibition of tumor metastases as well as of primary tumor growth. Simultaneous knock-down of Src and Stat3, and/or Myc exhibited the greatest effects resulting in substantial inhibition of primary tumor growth and metastasis.

**Conclusions/Significance:**

These findings demonstrate the effectiveness of simultaneous targeting of Src and the downstream signaling partners Stat3 and/or cMyc to inhibit the growth and oncogenic properties of a human cancer cell line. This knowledge may be very useful in the development of future therapeutic approaches involving targeting of specific genes products involved in tumor growth and metastasis.

## Introduction

Human breast cancer results from a combination of events and changes that alter the growth properties of breast epithelial cells. Some of these changes have been characterized to provide a clear contribution to the development and/or progression of the cancer and include overexpression of HER2/neu in about 20% of breast cancer [1], and hereditary mutations in BRCA1 or BRCA2 in approximately 5% of breast cancers [2]. Other alterations are less well defined in terms of their contribution to the final neoplastic phenotype, and include activation of Src, which has been shown in up to 30–70% of breast cancers by our lab and others [3]–[5].

**Figure 1:**
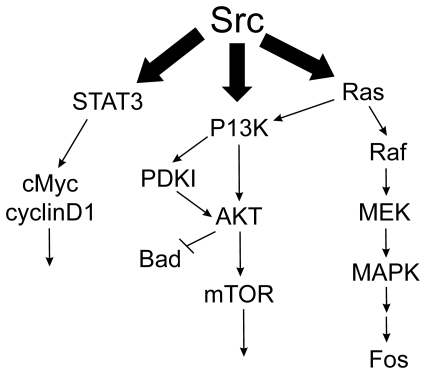
Src and some of the signaling pathways downstream of Src. Src regulates several downstream signaling pathways including the STAT3 pathway, the PI3K pathway, and the MAPK pathway.

Src is a non-receptor tyrosine kinase that can cause cellular transformation in cell culture and tumor formation in animals if its activity becomes elevated. Src's effects are thought to be mediated by activation of downstream signaling pathways including the mitogen-activated protein kinase (MAPK), the phosphatidylinositol 3-kinase (PI3K), and the signal transducer and activator of transcription 3 (STAT3) pathways (Fig. 1). Therefore, Src acts as a master control element, regulating many aspects of oncogenesis, since Src activation of these and other pathways can stimulate cell proliferation, motility, angiogenesis, invasion, and metastasis [6]–[8]. Src activity is elevated in several types of human cancers, including cancers of the breast, colon, ovary, prostate, and pancreas [3], [4], [9]–[12] and in melanomas [13]. In some breast cancer models, inhibition of Src activity suppresses the transformed phenotype of breast cancer cell lines [14] and restores tamoxifen sensitivity to tamoxifen-resistant breast cancer cell lines [15], suggesting it may be a useful target for therapy.

We wished to address whether Src and its downstream pathways might play a role in human breast cancer and whether targeting these pathways for suppression using short interfering RNA (siRNA) might have value as a future therapeutic. It was felt that Src was a good candidate for knock-down because: 1) elevation of Src activity has been implicated in the development and/or progression of human cancer, 2) efficient knock-down of Src is unlikely to cause impairment of normal cells, as Src knockout mice are viable [16]; and 3) it is unlikely that complete knock-down of Src would be required to elicit a cellular effect on cancer cells, as low levels of Src activity are present in most normal cell types.

As a model system, we chose to examine MDA-MB-435S, a highly metastatic cancer cell line that we previously had shown to possess high Src activity [3], [17], [18]. This cell line has been utilized in over 780 scientific papers as a model breast cancer cell line, but some controversy has arisen in the literature over the last few years regarding its classification [19]–[21], as it possesses some melanocytic characteristics. More recently, considerable evidence has been provided by several laboratories supporting the breast cancer origin of these cells [22], [23] as well as the classification of the MDA-MB-435S cells into the basal subset of human breast cancers that often express melanocyte-related genes [24], [25]. The MDA-MB-435S cells have also been characterized to fall within the claudin-low subtype of breast cancer cells which are enriched in epithelial-to-mesenchymal transition and stem-cell like features [26]. The basal subtype of human breast cancer is typically triple-negative (estrogen receptor, progesterone receptor, and HER2/neu negative), and as a consequence has a poor prognosis, as it is most often poorly responsive to many of the current treatment strategies [27]. Basal subtype tumors can also be particularly aggressive, and often more likely to recur than other subtypes of breast cancer. Therefore, it is very important that we develop alternate methods of treatment that target this particular subtype of cancer.

Cancer therapies targeting specific proteins are relatively new, and include antibodies (eg. Herceptin against HER2/neu), chemical inhibitors (Imatinib against Bcr/Abl), and now, siRNA. As compared to inhibitors targeting the protein products of various genes, siRNA shifts the focus of the targeting strategy from the protein that contributes to the malignancy to the mRNA that produces the protein. This is made possible by the use of siRNAs and their utilization of the RNA interference (RNAi) pathway to degrade specific cellular mRNAs [28], [29], thereby modifying protein expression levels and activities. This strategy provides the opportunity to pick key genes in the malignant process irrespective of whether it may be difficult to develop traditional pharmaceutical agents that target these proteins (ie. transcription factors such as Stat3). siRNAs also possess additional features which make them highly attractive as pharmaceuticals, including 1) their high degree of specificity, 2) the ease with which multiple siRNAs targeting a single proteins can be designed, thereby reducing the likelihood of drug resistance, and 3) the ability to simultaneously target two or more gene products. siRNAs are introduced into cultured cells directly by transfection or by utilizing plasmid or viral vectors, and similar techniques have been utilized in whole animals, where “naked” siRNA can be delivered to tissues/organs to treat certain disease conditions [30]. In humans, the administration of siRNA using various carrier molecules is in its early stages of testing, but promising early results have been obtained. With perhaps the greatest relevance to cancer treatment, investigators have now begun phase I clinical trials involving the systemic administration of siRNA (in targeted nanoparticles) to patients with solid cancers [31]. Their data demonstrate that the siRNA is delivered to the tumors and is capable of knocking down specific mRNAs, which is very encouraging news for laboratories currently developing siRNA-based therapeutics for human cancers.

We wished to test the feasibility of the direct application of siRNA complexed with lipids for the knock-down of Src, whose role in the maintenance of the complex phenotype of cancer is not clearly understood. It also seemed relevant to examine the effect of inhibiting more than a single signal transducer in the Src signaling pathway, or interacting pathways, through the use of other siRNAs in combination with Src siRNA. Direct application of siRNA avoids the use of viral-based vectors whose safety when used in humans is often currently under question. In this report, Src knock-down in combination with the knock-down of the downstream molecules STAT3 or cMyc is shown to result in a strong inhibition of the anchorage-independent growth, tumor growth, and metastasis of a human cancer cell line.

## Results

### siRNA targeting Src knocks down protein expression and affects the anchorage-independent growth of MDA-MB-435S cells

**Figure 2:**
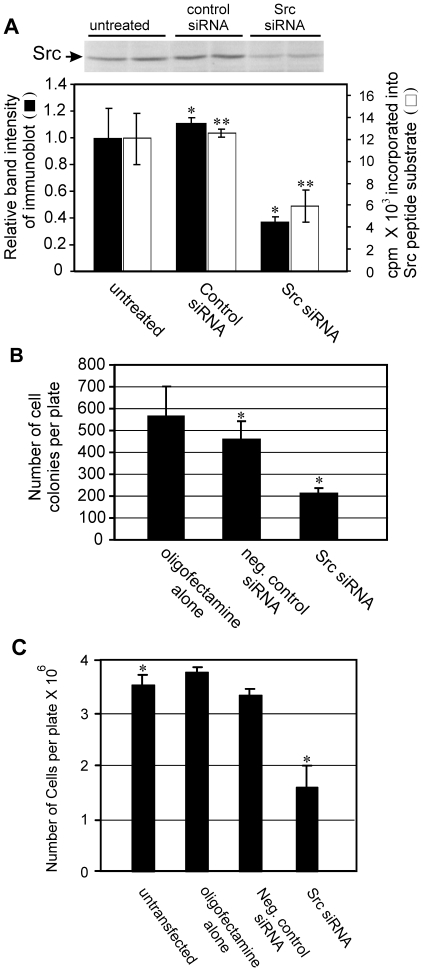
Src siRNA causes reductions in Src protein and activity and inhibits cell growth. MDA-MB-435S cells were left untreated or transfected with control or Src siRNA. 24 hours later, some cells were plated into soft agar or tissue culture dishes. After 48 hours, the remainder of the cells were processed for Src immunoblots or Src kinase activity. The results from the immunoblot and kinase assay were quantitated, the results are shown, and significant differences are indicated (* and **; p<0.05) (**A**). The cells in soft agar were grown for 14 days and colonies were quantitated (**B**) with significant differences indicated (*; p<0.05). The cells in monolayers were quantitated 5 days after plating and the significant differences indicated (*; p<0.01) (**C**). Each data point is the mean of duplicate (western and kinase) or triplicate (soft agar and monolayer) samples +/−1 S.D. and is representative of at least 3 independent experiments.

**Figure 3:**
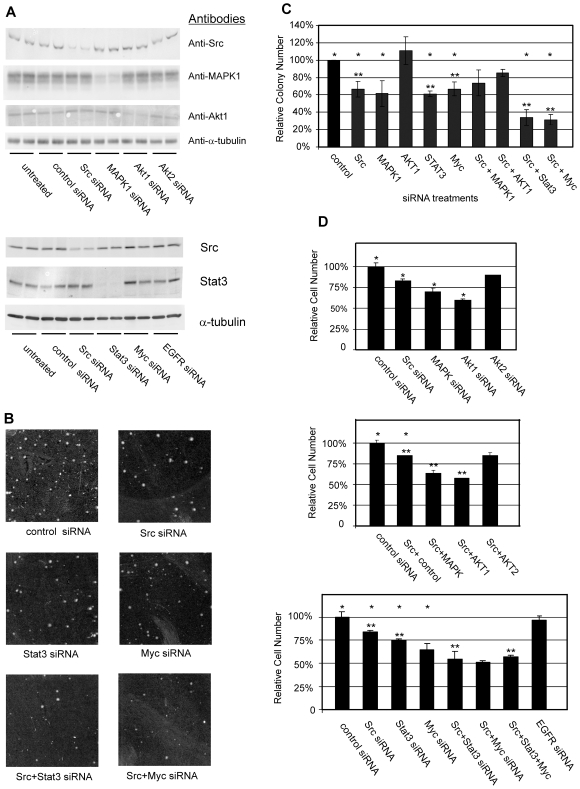
siRNA targeting various signaling molecules causes corresponding reductions in protein expression and inhibits cell growth. MDA-MB-435S cells were transfected with siRNA, and 24 hrs post transfection, cells were plated into soft agar or into tissue culture dishes. Cells to be analyzed by immunoblotting were lysed 60 hrs post-transfection and duplicate samples of each cell extract were analyzed (**A**). The cells in soft agar were grown for 14 days (**B**) and colonies larger than approximately 50 cells were quantitated (**C**). The cells in monolayer culture were quantitated 4 days after plating (**D**). Each growth condition was assayed in triplicate and is representative of at least 3 independent experiments. In C and D, comparisons were made between the group treated with control siRNA and various single targeting siRNA, and statistically significant differences are noted (*; p<0.05). In C and D, comparisons between singly and multiply transfected cells are made, and significant differences are noted (**; p<0.05).

Of the several siRNAs targeting Src that were designed and tested, the most effective one was used to knock down Src in the highly metastatic human breast cancer cell line MDA-MB-435S [17], a cell line in which Src activity is elevated [3] and potentially playing an important role in maintaining its neoplastic phenotype. Src siRNA caused a 62 or 67% reduction, respectively, in Src protein levels relative to either untransfected cells or cells transfected with a non-targeting control siRNA (significant differences with p<0.05; see methods and fig. legends for description of statistics for each figure) (Fig. 2A). The non-targeting control siRNA caused no significant change in Src levels relative to untrasnfected cells. This reduction by Src siRNA was accompanied by a corresponding reduction in Src kinase activity. The inhibitory effects typically lasted 4–5 days in rapidly dividing cells in culture (results not shown), and this duration was sufficient to carry out cell culture growth experiments. We noted that siRNAs targeting other gene products often resulted in 90–95% decreases in protein levels (see Fig. 3), suggesting that the relatively long (approximately 12–18 hrs) half-life of the cellular Src protein [32] may play a role in the effectiveness of the several Src siRNA we tested, which typically gave reductions of 50–80% (results not shown). In slowly growing cells and “in vivo”, siRNA silencing can be effective for greater than 21 days (unpublished observations and [33]).

Src siRNA was examined for its ability to alter certain growth characteristics of these tumor cells. The ability to suppress anchorage-independent growth in soft agar, a characteristic of transformed or cancer cells that is highly correlated with tumorigenicity [34], was initially examined. The Src siRNA was able to reduce the number of colonies in soft agar by 61% and 53% relative to either oligofectamine alone or negative controls siRNA treated cells, respectively (Fig. 2B). Treatment with Src siRNA also affected anchorage-dependent growth and resulted in a 50% reduction in cell number in comparison to untreated cells 5 days post transfection (Fig. 2C).

### Src knock-down combined with knock-down of STAT3 or cMyc augments the effects on anchorage-independent and -dependent growth

The cell growth effects of Src siRNA alone were further examined to see if they could be augmented by combining the Src siRNA with siRNAs that target signaling pathways that are either downstream of Src or that interact with the Src signal transduction cascade. siRNAs targeting proteins in several signaling pathways including MAPK1, Akt1, cMyc, and STAT3 were examined for their ability to modify cell growth.

These siRNAs were initially examined for their ability to reduce their corresponding target proteins. Western blots demonstrated that these siRNAs could significantly reduce protein expression levels of their corresponding proteins (Fig. 3A). Several (2–4) target sequences in each targeted protein were tested for gene knockdown, and the most potent sequence was selected for further use (results not shown). The effects of the siRNAs appeared specific to their targeted protein. The degree of reduction varied somewhat between the different siRNAs, but these siRNAs were capable of reducing protein levels by 60–95% (quantitated blots, results not shown). Akt2 or EGFR proteins were undetectable in this cell line.

MDA-MB-435S cells were transfected with a single siRNA or various siRNAs in combination with Src siRNA and the effects on growth in soft agar were examined. In contrast to the experimental protocols utilized in previous figures, the cells were transfected only once, which resulted in slightly lower transfection efficiencies and slightly lowered growth effects, but simplified the procedure and reduced the usage of siRNA reagents. Of the siRNAs tested, those targeting Src, STAT3, and cMyc were among the most effective in inhibiting anchorage-independent colony formation in soft agar relative to the control siRNA group. Src siRNA caused a 35% reduction, and Stat and cMyc caused reductions of 38% and 35%, respectively, under these conditions (Fig. 3B and C). Akt1 appeared to have little or no effect on growth in soft agar. However, simultaneous targeting of Src and STAT3, or Src and cMyc produced a much greater effect, resulting in a 65–70% reduction in colony number relative to control siRNA, and this exceeded the inhibitory effects observed with any single siRNA (significant differences between single siRNA treatments and dual siRNA treatments).

The ability of the siRNAs individually and in combination with Src siRNA to cause changes in cell growth in monolayer culture was examined. When the cells were treated with the different individual siRNAs, it was observed that the Src, MAPK1, Akt1, STAT3, and cMyc siRNA reduced the cell number relative to control siRNA-treated cells by 20% to 40%, while siRNAs for Akt2 and EGFR did not exhibit significant effects on monolayer cell growth (Fig. 3D). When Src siRNA was combined with the other siRNAs, additional reductions in growth over the single siRNAs alone using either cMyc siRNA or STAT3 siRNA were observed (40–50% reduction in cell number relative to control siRNA-treated plates) (Src and Stat3 combined were significantly different than either Src or Stat3 alone; but the differences between Myc alone and Myc combined with Src or Stat3 or both, were not).

### Src and STAT3 knock-down reduce tumor formation in NOD/SCID mice

siRNA treatment of the MDA-MB-435S cells was examined to determine if it would affect their ability to form tumors in NOD/SCID mice. Our laboratory (results not shown) and others have previously shown that MDA-MB-435 cells form primary tumors when implanted either subcutaneously on the back or into the mammary fat pad of mice and metastasize to sites such as the lymph nodes and lungs [18], [35], [36]. To facilitate the identification of the implanted tumor cells, cells that stably expressed GFP protein were utilized. siRNA transfected cells (control or Src+STAT3 siRNA) were implanted into the mammary fat pad (4 mice/group) and the mice were observed for development of palpable tumors at the site of implantation.

**Figure 4:**
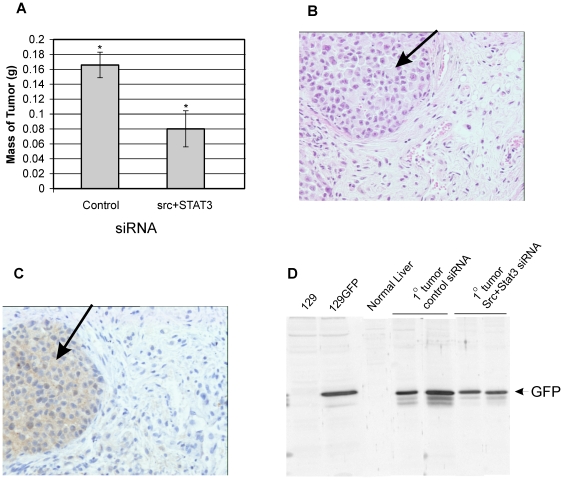
Src+STAT3 siRNA reduces tumor formation in NOD/SCID mice. 1×10^6^ cells transfected with control (mice 1–4) or Src+STAT3 siRNAs (mice 5–8) were implanted into the mammary fat pad of mice. After 66 days, the tumors were excised and weighed (significant differences are indicated (*; p<0.05)(**A**). Sections of the tumor were stained (**B**) or subjected to immunostaining with anti-GFP antibody (**C**). Malignant cells are indicated by an arrow. A portion of each tumor was homogenized with RIPA buffer and immunoblotted with anti-GFP antibodies (**D**).

On day 66, the mice were euthanized and examined. Primary tumors were observed at the site of implantation. The tumors were excised and weighed. The average weight of the tumors from the control siRNA mice was 0.167 g, compared to 0.080 g in the Src+STAT3 siRNA group (Fig. 4A). Microscopic examination detected the presence of cancer cells in the tumor (Fig. 4B) and the presence of the MDA-MB-435S cells was confirmed by immunostaining (Fig. 4C) and immunoblotting (Fig. 4D) for GFP. These results demonstrated that transfection with Src+STAT3 siRNA was able to significantly reduce but not eliminate the ability of the cells to form tumors in the mice.

**Figure 5:**
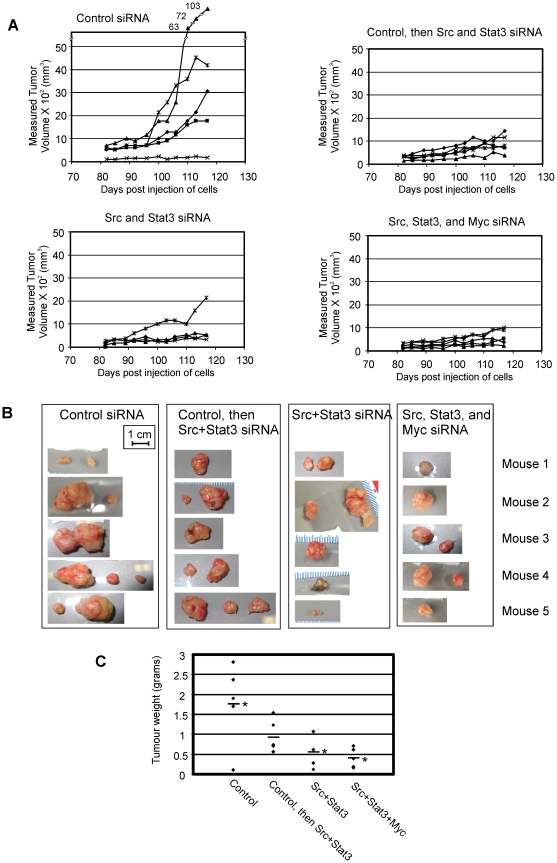
Injection of siRNA/oligofectamine complexes into mouse tumors inhibits tumor growth. MDA-MB-435S cells were implanted in the mammary fat pad of SCID/NOD mice. Two days post-implantation, the mice were divided into 3 groups (one group of 10 control siRNA-injected mice and two groups of 5 targeted siRNA mice) and the groups received twice weekly injections of either control, Src+STAT3, or Src+STAT3+Myc siRNA into the site of the cell implantation/tumor. 47 days post-implantation, 5 mice in the control group were switched to Src+STAT3 siRNA injections for the remainder of the experiment (Control, then Src+STAT3 group). Tumor growth was monitored and the results are shown (**A**), with each treatment group shown in a single panel, and individual mouse tumors represented by connected data points. 126 days post-implantation, the primary tumor(s) were excised (**B**) and weighed. The tumor weights for each mouse (data points) and the average tumor weight for each group (horizontal bar) is shown (**C**). Tumor weight data was analyzed by Analysis of Variance and Tukey's pairwise comparison, with significant differences between the control group and two of the treatment groups noted ( *p<0.05).

We then sought to determine whether siRNA might be able to suppress the long-term post-implantation growth of tumor cells through experiments involving direct intra-tumoral injection of siRNAs. MDA-MB-435S cells were implanted into the mammary fat pad, and starting two days post-implantation, the mice were treated with control, Src+STAT3, or Src+STAT3+Myc siRNA. siRNA complexed with lipid-based transfection reagents have previously been show to be effective at delivering siRNA to cells in whole animal models [37] and results from our laboratory (unpublished observation) showed an approximately 30% reduction in luciferase signal following a single injection of lipid-complexed luciferase siRNA into a 3 mm diameter luciferase-expressing tumor. After allowing the tumors to grow to approximately 6–7 mm in diameter (day 47 post-implantation), half the mice receiving the control siRNA were changed to injections of Src+STAT3 siRNA. Between 80 days up until the end of the experiment, the control group showed more rapid tumor growth than the other 3 injection groups, and had the largest average tumor size of 3899 mm^3^ (Fig. 5A). The other 3 groups, including mice changed from control to Src+STAT3 siRNA, the Src+STAT3 group, and the Src+STAT3+Myc group had smaller average tumor sizes of 892, 878, and 610 mm^3^, respectively. The experiment was ended on day 126, the tumors were examined, and weighed (Fig. 5B and C). The control group had the largest average tumor weight (1.78 g), followed by the group that received control siRNA before receiving Src+STAT3 siRNA (0.96 g), the Src+STAT3 group (0.52 g), and the Src+STAT3+Myc group (0.41 g), and these measurements agreed well with the tumor size measurements. These results demonstrated that siRNA is capable of inhibiting tumor growth locally when injected into the site of the tumor in whole animal models. They also illustrate a potentially useful method to increase the effectiveness of siRNAs to elicit cellular effects through the use of combinations of siRNAs to target key components of one or more signaling pathways.

### Inhibition of tumor metastasis

**Figure 6:**
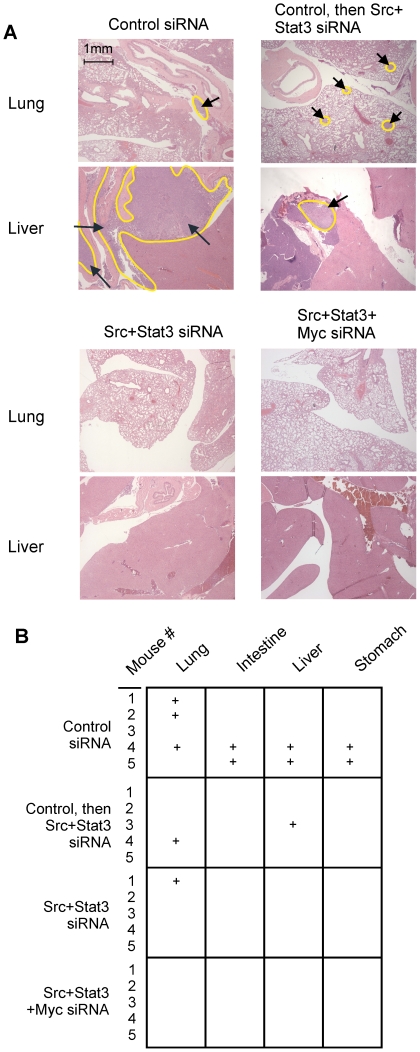
Injection of siRNA/oligofectamine complexes into mouse tumors inhibits tumor metastasis. Mice treated in fig. 5 were examined grossly and microscopically for the presence of tumor metastasis and representative pictures (**A**) (tumors indicated with yellow outline and arrows) are shown. A summary of results from gross and microscopic examination was compiled (**B**) with the presence to metastatic tumor growth indicated (+).

Partial dissection of each mouse was performed to examine for the presence of tumor metastases, followed by microscopic examination for the presence of tumor cells. Representative microscopic views of the lung and liver are shown in Fig. 6A, and summarized results from the combined gross and microscopic examination in Fig. 6B. 4 of 5 mice in the control group had metastatic tumor infiltration in various organs, including the lung, liver, stomach, and intestine. Three mice in this group had grossly-visible tumors, including two with abdominal tumors and one with tumor nodules in the lungs. 3 of 5 mice in the group changed from control to Src+STAT3 siRNA had metastases that were detectable only after microscopic examination of the lung and liver. Grossly- and microscopically-visible metastasis developed in the lung of only 1 of 5 mice in the Src+STAT3 group and in none of the 5 mice in the Src+STAT3+Myc group. These results show that knock-down of Src and STAT3 can reduce the appearance of metastasis and suggest that not only are Src and STAT3 important in the growth of the primary tumor, but may have a role in the metastatic process.

### Src and STAT3 siRNA induce apoptosis in treated cells

**Figure 7:**
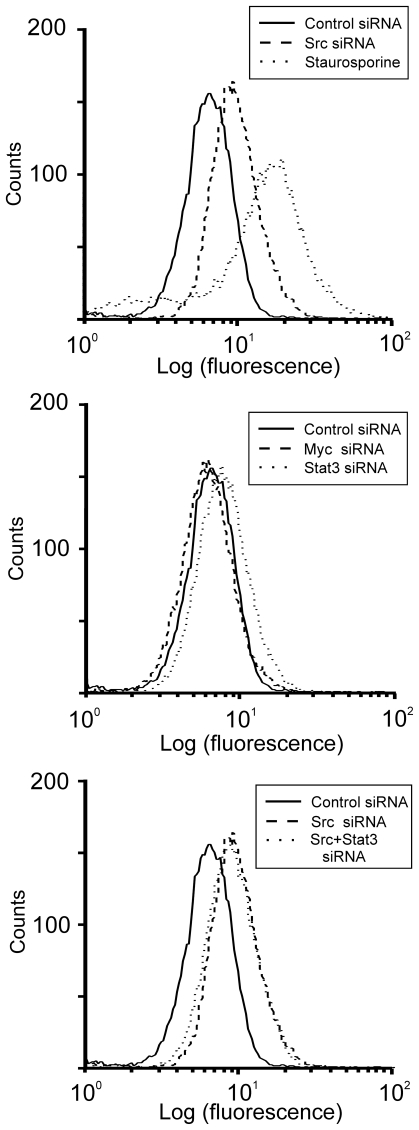
Src siRNA causes apoptosis of MDA-MB-435S cells. MDA-MB-435S cells were transfected with siRNA or treated with 1 uM staurosporine. 48 hours post-treatment, the cells were collected and assayed for apoptosis using the TUNEL assay. Results are expressed with log(fluorescence) on the horizontal axis and cell counts on the vertical axis and is representative of triplicate experiments.

The siRNAs may have affected cell growth through one or several mechanisms, including the stimulation of apoptosis, and this particular possibility was assessed using the TUNEL assay (Fig. 7). Staurosporine, a potent pro-apoptotic agent, caused an increase in apoptotic cells (upper panel, dotted line) compared to control cells (solid line), as evidenced by an increased incorporation of fluorescein-labeled dUTP into DNA of the treated cell population (rightward shift of curve). Src siRNA was also found to cause increased apoptosis in the MDA-MB-435S cells, (upper panel, dashed line), as compared to control cells (solid line). STAT3 siRNA caused a smaller, but significant shift in the cell population (middle panel). Myc siRNA caused no significant change. Combining Src plus STAT3 siRNA (lower panel, dotted line) did not increase TUNEL staining over that seen with Src siRNA alone.

## Discussion

Our results indicate that knock-down of Src, and its downstream signaling partners STAT3 and cMyc, using siRNA in the human cancer cell line MDA-MB-435S, have a dramatic effect on its neoplastic properties. Importantly, in a mouse model, the siRNA treatments were highly successful at reducing the formation of tumor metastasis, and this was accompanied by reduced growth of the primary tumor. This is of particular interest, because currently, metastatic forms of human malignancies are particularly difficult to treat. In addition, MDA-MB-435S is a triple-negative cell line (estrogen receptor, progesterone receptor and HER2 negative) that belongs to the basal type of breast cancers [38], and that are typically unresponsive to receptor-targeted treatments.

Our results indicate that knock-down of Src using siRNA in the MDA-MB-435S cells has a significant effect on the anchorage-independent growth characteristics of the cells, an important hallmark of cell transformation, and to a lesser extent, reduced the growth rate in monolayer cultures. Src is upstream of several important pathways that have been implicated in the neoplastic phenotype [39], and it is likely that targeting Src for knock-down also has effects on these downstream signaling pathways, including the Ras/MAPK, AKT/PKB, and STAT3/Myc pathways. The suppression of anchorage-independent growth was substantial but not complete. This probably reflects the complex nature of the transforming defect that is present as well as our inability to fully suppress Src activity. siRNAs against other single targets, including STAT3 and cMyc, also demonstrated similar abilities to inhibit anchorage-independent growth. STAT3 and cMyc are downstream mediators of Src (see Fig. 2), and as such, might be expected to show somewhat similar inhibitory patterns as Src. Because the effects of the siRNAs on anchorage-independent growth seemed to correlate with their effects on tumor growth in our mouse model, this suggests that growth in soft agar might be an effective method of screening the responsiveness of cells to larger/different panels of siRNAs.

Targeting two or more growth signaling molecules may serve to inhibit multiple parallel pathways which are collectively important for cell growth or may inhibit a single pathway more efficiently by suppressing more than one mediator along the pathway. This second mechanism may be of particular relevance where siRNA-mediated protein knock-down is not 100% efficient, which is common when utilizing siRNAs. It may be easier and/or more effective to knock-down two or more genes that lie along the same pathway for an additive effect than to attempt to suppress the pathway by targeting a single gene product.

Most of the effects we observed upon cell growth when combining siRNAs were additive or less than additive under the limited number of growth conditions tested here. Additive inhibition can occur within the same pathway or in parallel pathways, and the literature supports the likelihood that a significant component of the inhibition we observed with Src, Stat3, and Myc comes from inhibiting this single pathway (see Fig. 1). Based upon our understanding that there are likely multiple positive signaling inputs (signaling pathway crosstalk) along the Src, Stat3, Myc pathway (eg. Stat3 can also be activated by Jak2 in addition to Src), it seems desirable to suppress the pathway at multiple levels to achieve a maximal effect under a variety of cellular conditions.

Combining two or more siRNAs has the potential to greatly expand the biological potency of RNAi. It has the major advantage over single protein targeting strategies that if single protein knock-down doesn't give the desired response, combinations of proteins can be targeted. Libraries of RNAi reagents are currently available and screening methods allow the examination of many different combinations of siRNAs. Delivery of multiple siRNAs is no different than that for single siRNAs, and many of the complex “in vivo” drug interactions that occur between traditional pharmaceuticals do not apply to these agents, thereby facilitating their future use in humans.

In the mouse tumor models, cells pretreated with Src and STAT3 siRNA by transfection or treated by direct injection of siRNA into the tumor showed a strong reduction in the ability to form primary tumors. No overt toxic effects of the siRNA injections were observed using the dosages utilized in this study. It is possible that higher dosages of siRNA may be more efficacious. Further improvements in technology and in the stability of siRNA are also likely to make this approach more efficient. In addition, it was noted that siRNA injections into the primary tumor have a major effect to inhibit the development of distant metastasis in organs such as the lung and liver. In our experiments, this was most evident in the group of mice treated with Src+STAT3+Myc siRNAs, where there was complete blockage of metastasis. Because the primary tumors were significantly smaller in the treatment vs control group, it is possible that at least part of this effect may be due to a reduction in the malignant cell number of the primary tumor, with a corresponding reduction in the likelihood of tumor spread. Alternatively, it is possible that Src+STAT3 siRNA or Src+STAT3+Myc may cause changes in the invasive properties of the cells in the environment of the primary tumor that reduces their metastatic potential. Src is known to promote metastasis through its effects on vascular endothelial growth factor synthesis [40], angiogenesis [7], [41], production of metalloproteases [42], cell adhesion [43], and cell motility [44]. In this regard, our lab has demonstrated that Src promotes the destabilization of the Von Hippel-Lindau tumor suppressor protein that negatively regulates vascular endothelial growth factor synthesis [45]. STAT3 and cMyc have also been implicated in both primary tumor growth as well as tumor metastasis [46]–[48]. There is also a more remote possibility that the siRNA spread beyond the primary tumor to affect metastatic cells at remote sites.

RNAi is an important tool for the study of signaling pathways as well as a potential therapeutic agent for the suppression of aberrantly expressed and/or mutated gene products as are often found in human cancers. siRNA and short-hairpin RNA targeted against oncogenes such as K-RAS^V12^ [49], Twist [50], and others can suppress the neoplastic phenotype of cancer cells. RNAi has been demonstrated to be more effective than other gene targeting approaches such as anti-sense RNA and anti-sense oligonucleotides [51], [52], and studies utilizing direct intravenous application of siRNA to target genes such as FAS for the treatment of fulminant hepatitis [53] support the usefulness of direct application of siRNA to control the expression of target proteins in cancer and other diseases. The functional stability of conventional siRNAs in whole animals has been reported to span at least 14–21 days [33] and the introduction of chemical modifications such as 2′-O methyl groups may extend the duration of their effectiveness [54]. It will be important to examine alternate modes of delivery, as many naturally-occurring tumors are not readily accessible. Along this line, we and others are investigating delivery vehicles that would allow targeted delivery of siRNA, such as cell-targeting peptides incorporated into siRNA-containing nanoparticles. Once methods of delivery into whole organisms are perfected, siRNA could prove a powerful adjunct and/or substitute for current treatment protocols for several diseases, including many forms of cancer.

## Materials and Methods

### Ethics Statement

All animals used in this study were housed and received care according to the University of Calgary Animal Care Committee guidelines. Prior to commencement of this study, procedures were reviewed and approved by the University of Calgary Animal Care Committee, Protocol M07108, and are in accordance with the principles outlined in “The Guide to the Care and Use of Experimental Animals” by the Canadian Council on Animal Care.

### siRNA molecules

RNA for the Src, STAT3, cMyc, and control siRNA was synthesized by the University of Calgary Core DNA/RNA facility (Dr. R. Pon) and annealed in RNA buffer (6 mM Hepes pH 7.5, 20 mM KCl, and 0.2 mM MgCl_2_) to form double stranded siRNA (20 µM) with 3′ 2 nucleotide dTdT or UU overhangs. Several siRNAs were screened for each protein targeted and the siRNA giving the best knockdown was chosen for use. The control siRNA target sequence was AATTCTCCGAACGTGTCACGT. The Src siRNA target sequence was TGTTCGGAGGCTTCAACTCCT. The cMyc siRNA target sequences were GCTTCACCAACAGGAACTA, AAACATCATCATCCAGGAC, CCTGAGCAATCACCTATGA, AACGATTCCTTCTAACAGA, and ACGACGAGACCTTCATCAA (siRNA mixed in equimolar amounts). The STAT3 siRNA target sequence was GGCGTCCAGTTCACTACTA. siRNAs targeting Akt1, Akt2, the epidermal growth factor receptor (EGFR), and MAPK1 were Smartpools from Upstate Biotech. Inc (New York, USA).

### Antibodies

A hybridoma producing MAb327 anti-Src antibody [55] was a kind gift from J. Brugge. Ab-1 anti-α-tubulin antibody was from Oncogene Research Products. Anti-green fluorescent protein (GFP) antibody was from Santa Cruz (California, USA). Antibodies against MAPK, cMyc, STAT3, Akt1, and Akt2 were from Upstate Biotech. Inc. H9B4 antibody against the EGFR has been previously described [56].

### Transfection of Cultured Cells

MDA-MB-435S cells from the American Type Culture Collection were maintained in Dulbecco's modified Eagle's medium (DMEM) containing 10% fetal bovine serum. Cells seeded in 24 well dishes were transfected with siRNAs (200 nM) using Oligofectamine (Invitrogen) according to the manufacturer's protocol. 24 hours later, the cells were detached using trypsin, and replated in 12 well tissue culture dishes. In some cases, the cells were retransfected the following day to ensure a high percentage of the cells became transfected.

### Cell lysis, immunoblotting of proteins, and measurement of kinase activity

Cells were lysed using RIPA buffer and processed for immunoblotting [57] or kinase activity [58] as previously described.

### Cell Growth and Soft agar colony forming assay

Cells to be grown as monolayers were replated into 10 cm tissue culture dishes in 2.5% fetal bovine serum/2.5% calf serum in DMEM at a density of 1.5×10^5^ cells/dish. The medium was changed once 3 days after plating and the cell number was quantitated using a Beckman Z1 Particle Counter 5 days after plating. Cells for soft agar assays were suspended at a density of 2.35×10^4^ cells/dish in soft agar and treated as previously described [57]. Images of the plates were made using a Microtek scanner. Statistical analysis of data was performed using Tukey's multiple comparison test and unpaired t-tests.

### Terminal Deoxynucleotidyl Transferase dUTP Nick End Labeling (TUNEL) Assay

Cells were detached using trypsin, washed twice with phosphate buffered saline (PBS), and fixed for 20 min. in 1.5% paraformaldehyde in PBS at 4°C with gentle mixing. The cells were then washed twice with PBS, suspended in 70% ethanol at 4°C, and stored at −20°C until assayed. When assayed, the cells were washed twice with PBS and then resuspended in 50 µl of Tunel assay buffer consisting of 100 mM potassium cacodylate (pH 7.2), 2 mM CoCl_2_, 0.2 mM dithiotheitol, 3.3 nM fluorescein-conjugated dUTP, 13 nM dATP and 11 units of terminal deoxynucleotidyl transferase for 90 minutes at 37°C. The cells were then washed twice with PBS and analyzed by FACS sorting

### Tumor Formation in NOD/SCID Mice

1×10^6^ cells were implanted by injection into the mammary fat pad of 5–7 week old NOD/SCID mice (Jackson Laboratory). In some experiments (as noted), the cells were initially transfected with control siRNA, Src+STAT3 siRNAs, or Src+STAT3+cMyc siRNAs. The cells were then detached and resuspended in 100 µl phenol red-free DMEM/PBS (50/50) for implantation. In some experiments, the mice also received twice weekly injections of siRNA complexed with Oligofectamine into the tumor, or into the site of tumor implantation (prior to appearance of a visible tumor). Each siRNA injection consisted of 1.2 nmoles of siRNA (approx. 0.8 mg/kg) complexed with 13 µl of Oligofectamine in a final volume of 110 µl of Optimem I medium (Invitrogen). Tumor diameters were measured every 3–4 days using calipers and the measurements were converted to tumor volumes using the formula for a sphere. At the end of the experiment, tumors were excised, weighed, and the data analyzed using Tukey's multiple comparison tests and unpaired t-tests.

### Tissue fixation, sectioning, and staining

Tumor or tissue samples were fixed in phosphate-buffered 4% formaldehyde and processed by standard histological methods. 5 µm paraffin sections were stained with hematoxylin and eosin or were subjected to immunostaining with anti-GFP antibody. Briefly, sections were deparaffinized and treated with Target Retrieval Solution (Dako) according to the manufacturer's protocol. The sections were then stained using a Dakocytomation Autostainer (Model LV-1) using a 1∶600 dilution of anti-GFP antibody (Santa Cruz). The staining was visualized using horseradish peroxidase-conjugated goat anti-rabbit antibody, followed by 3,3-diaminobenzidine. Counterstaining was with Mayer's Hematoxylin and Bluing solution (Harleco).

## References

[1] Slamon DJ, Clark GM, Wong SG, Levin WJ, Ullrich A (1987). Human breast cancer: correlation of relapse and survival with amplification of the HER-2/neu oncogene.. Science.

[2] Fackenthal JD, Olopade OI (2007). Breast cancer risk associated with BRCA1 and BRCA2 in diverse populations.. Nat Rev Cancer.

[3] Egan C, Pang A, Durda D, Cheng HC, Wang JH (1999). Activation of Src in human breast tumor cell lines: elevated levels of phosphotyrosine phosphatase activity that preferentially recognizes the Src carboxy terminal negative regulatory tyrosine 530.. Oncogene.

[4] Ottenhoff-Kalff AE, Rijksen G, van Beurden EA, Hennipman A, Michels AA (1992). Characterization of protein tyrosine kinases from human breast cancer: involvement of the c-src oncogene product.. Cancer Res.

[5] Rosen N, Bolen JB, Schwartz AM, Cohen P, Deseau V (1986). Analysis of pp60c-src protein kinase activity in human tumor cell lines and tissues.. J Biol Chem.

[6] Ishizawar R, Parsons SJ, Ishizawar R, Parsons SJ (2004). c-Src and cooperating partners in human cancer. [Review] [63 refs].. Cancer Cell.

[7] Trevino JG, Summy JM, Lesslie DP, Parikh NU, Hong DS (2006). Inhibition of SRC expression and activity inhibits tumor progression and metastasis of human pancreatic adenocarcinoma cells in an orthotopic nude mouse model.. Am J Pathol.

[8] Myoui A, Nishimura R, Williams PJ, Hiraga T, Tamura D (2003). C-SRC tyrosine kinase activity is associated with tumor colonization in bone and lung in an animal model of human breast cancer metastasis.. Cancer Res.

[9] Irby RB, Mao W, Coppola D, Kang J, Loubeau JM (1999). Activating SRC mutation in a subset of advanced human colon cancers.. Nat Genet.

[10] Lutz MP, Esser IB, Flossmann-Kast BB, Vogelmann R, Luhrs H (1998). Overexpression and activation of the tyrosine kinase Src in human pancreatic carcinoma.. Biochem Biophys Res Commun.

[11] Wiener JR, Nakano K, Kruzelock RP, Bucana CD, Bast RC (1999). Decreased Src tyrosine kinase activity inhibits malignant human ovarian cancer tumor growth in a nude mouse model.. Clin Cancer Res.

[12] Chang YM, Bai L, Liu S, Yang JC, Kung HJ (2008). Src family kinase oncogenic potential and pathways in prostate cancer as revealed by AZD0530.. Oncogene.

[13] Barnekow A, Paul E, Schartl M (1987). Expression of the c-src protooncogene in human skin tumors.. Cancer Res.

[14] Ishizawar RC, Tice DA, Karaoli T, Parsons SJ (2004). The C terminus of c-Src inhibits breast tumor cell growth by a kinase-independent mechanism.. J Biol Chem.

[15] Chu I, Sun J, Arnaout A, Kahn H, Hanna W (2007). p27 phosphorylation by Src regulates inhibition of cyclin E-Cdk2.. Cell.

[16] Soriano P, Montgomery C, Geske R, Bradley A (1991). Targeted disruption of the c-src proto-oncogene leads to osteopetrosis in mice.. Cell.

[17] Cailleau R, Olive M, Cruciger QVJ (1978). Long-Term Human Breast Carcinoma Cell Lines of Metastatic Origin - Preliminary Characterization.. In Vitro-Journal of the Tissue Culture Association.

[18] Price JE (1996). Metastasis from human breast cancer cell lines.. Breast Cancer Res Treat.

[19] Rae JM, Creighton CJ, Meck JM, Haddad BR, Johnson MD (2007). MDA-MB-435 cells are derived from M14 melanoma cells–a loss for breast cancer, but a boon for melanoma research.. Breast Cancer Res Treat.

[20] Ross DT, Scherf U, Eisen MB, Perou CM, Rees C (2000). Systematic variation in gene expression patterns in human cancer cell lines.. Nat Genet.

[21] Sellappan S, Grijalva R, Zhou X, Yang W, Eli MB (2004). Lineage infidelity of MDA-MB-435 cells: expression of melanocyte proteins in a breast cancer cell line.. Cancer Res.

[22] Chambers AF (2009). MDA-MB-435 and M14 cell lines: identical but not M14 melanoma?. Cancer Res.

[23] Hollestelle A, Schutte M (2009). Comment Re: MDA-MB-435 and M14 cell lines: identical but not M14 Melanoma?. Cancer Res.

[24] Montel V, Suzuki M, Galloy C, Mose ES, Tarin D (2009). Expression of melanocyte-related genes in human breast cancer and its implications.. Differentiation.

[25] Zhang Q, Fan H, Shen J, Hoffman RM, Xing HR (2010). Human breast cancer cell lines co-express neuronal, epithelial, and melanocytic differentiation markers in vitro and in vivo.. PLoS One.

[26] Prat A, Parker JS, Karginova O, Fan C, Livasy C (2010). Phenotypic and molecular characterization of the claudin-low intrinsic subtype of breast cancer.. Breast Cancer Res.

[27] Rakha EA, Ellis IO (2009). Triple-negative/basal-like breast cancer: review.. Pathology.

[28] Elbashir SM, Harborth J, Lendeckel W, Yalcin A, Weber K (2001). Duplexes of 21-nucleotide RNAs mediate RNA interference in cultured mammalian cells.. Nature.

[29] Caplen NJ, Parrish S, Imani F, Fire A, Morgan RA (2001). Specific inhibition of gene expression by small double-stranded RNAs in invertebrate and vertebrate systems.. Proc Natl Acad Sci U S A.

[30] Palliser D, Chowdhury D, Wang QY, Lee SJ, Bronson RT (2006). An siRNA-based microbicide protects mice from lethal herpes simplex virus 2 infection.. Nature.

[31] Davis ME, Zuckerman JE, Choi CH, Seligson D, Tolcher A (2010). Evidence of RNAi in humans from systemically administered siRNA via targeted nanoparticles.. Nature.

[32] Hakak Y, Martin GS (1999). Ubiquitin-dependent degradation of active Src.. Curr Biol.

[33] Bartlett DW, Davis ME (2006). Insights into the kinetics of siRNA-mediated gene silencing from live-cell and live-animal bioluminescent imaging.. Nucleic Acids Res.

[34] Montesano R, Drevon C, Kuroki T, Saint VL, Handleman S (1977). Test for malignant transformation of rat liver cells in culture: cytology, growth in soft agar, and production of plasminogen activator.. J Natl Cancer Inst.

[35] Lebedeva S, Bagdasarova S, Tyler T, Mu X, Wilson DR (2001). Tumor suppression and therapy sensitization of localized and metastatic breast cancer by adenovirus p53.. Hum Gene Ther.

[36] Tofani S, Barone D, Peano S, Ossola P, Ronchetto F (2002). Anticancer activity by magnetic fields: Inhibition of metastatic spread and growth in a breast cancer model.. Ieee Transactions on Plasma Science.

[37] Yano J, Hirabayashi K, Nakagawa S, Yamaguchi T, Nogawa M (2004). Antitumor activity of small interfering RNA/cationic liposome complex in mouse models of cancer.. Clin Cancer Res.

[38] Hollestelle A, Nagel JH, Smid M, Lam S, Elstrodt F (2009). Distinct gene mutation profiles among luminal-type and basal-type breast cancer cell lines.. Breast Cancer Res Treat.

[39] Bowman T, Broome MA, Sinibaldi D, Wharton W, Pledger WJ (2001). Stat3-mediated Myc expression is required for Src transformation and PDGF-induced mitogenesis.. Proc Natl Acad Sci U S A.

[40] Jiang BH, Agani F, Passaniti A, Semenza GL (1997). V-SRC induces expression of hypoxia-inducible factor 1 (HIF-1) and transcription of genes encoding vascular endothelial growth factor and enolase 1: involvement of HIF-1 in tumor progression.. Cancer Res.

[41] Trevino JG, Summy JM, Gray MJ, Nilsson MB, Lesslie DP (2005). Expression and activity of SRC regulate interleukin-8 expression in pancreatic adenocarcinoma cells: implications for angiogenesis.. Cancer Res.

[42] Noritake H, Miyamori H, Goto C, Seiki M, Sato H (1999). Overexpression of tissue inhibitor of matrix metalloproteinases-1 (TIMP-1) in metastatic MDCK cells transformed by v-src.. Clin Exp Metastasis.

[43] Matsuyoshi N, Hamaguchi M, Taniguchi S, Nagafuchi A, Tsukita S (1992). Cadherin-mediated cell-cell adhesion is perturbed by v-src tyrosine phosphorylation in metastatic fibroblasts.. J Cell Biol.

[44] Sakamoto M, Takamura M, Ino Y, Miura A, Genda T (2001). Involvement of c-Src in carcinoma cell motility and metastasis.. Jpn J Cancer Res.

[45] Chou MTH, Anthony J, Bjorge JD, Fujita DJ (2010). The von Hippel–Lindau Tumor Suppressor Protein Is Destabilized by Src.. Genes & Cancer.

[46] Scholz A, Heinze S, Detjen KM, Peters M, Welzel M (2003). Activated signal transducer and activator of transcription 3 (STAT3) supports the malignant phenotype of human pancreatic cancer.. Gastroenterology.

[47] Ling X, Arlinghaus RB (2005). Knockdown of STAT3 expression by RNA interference inhibits the induction of breast tumors in immunocompetent mice.. Cancer Res.

[48] Wang YH, Liu S, Zhang G, Zhou CQ, Zhu HX (2005). Knockdown of c-Myc expression by RNAi inhibits MCF-7 breast tumor cells growth in vitro and in vivo.. Breast Cancer Res.

[49] Brummelkamp TR, Bernards R, Agami R (2002). Stable suppression of tumorigenicity by virus-mediated RNA interference.. Cancer Cell.

[50] Yang J, Mani SA, Donaher JL, Ramaswamy S, Itzykson RA (2004). Twist, a master regulator of morphogenesis, plays an essential role in tumor metastasis.. Cell.

[51] Arens N, Gandhari M, Bleyl U, Hildenbrand R (2005). In vitro suppression of urokinase plasminogen activator in breast cancer cells–a comparison of two antisense strategies.. Int J Oncol.

[52] Miyagishi M, Hayashi M, Taira K (2003). Comparison of the suppressive effects of antisense oligonucleotides and siRNAs directed against the same targets in mammalian cells.. Antisense Nucleic Acid Drug Dev.

[53] Song E, Lee SK, Wang J, Ince N, Ouyang N (2003). RNA interference targeting Fas protects mice from fulminant hepatitis.. Nat Med.

[54] Dykxhoorn DM, Lieberman J (2006). Running interference: prospects and obstacles to using small interfering RNAs as small molecule drugs.. Annu Rev Biomed Eng.

[55] Lipsich LA, Lewis AJ, Brugge JS (1983). Isolation of monoclonal antibodies that recognize the transforming proteins of avian sarcoma viruses.. J Virol.

[56] Bjorge JD, Kudlow JE (1987). Epidermal growth factor receptor synthesis is stimulated by phorbol ester and epidermal growth factor. Evidence for a common mechanism.. J Biol Chem.

[57] Zhu S, Bjorge JD, Fujita DJ (2007). PTP1B contributes to the oncogenic properties of colon cancer cells through Src activation.. Cancer Res.

[58] Bjorge JD, Pang A, Fujita DJ (2000). Identification of protein-tyrosine phosphatase 1B as the major tyrosine phosphatase activity capable of dephosphorylating and activating c-Src in several human breast cancer cell lines.. J Biol Chem.

